# The role of IL-6 in atherosclerosis: a narrative review

**DOI:** 10.3389/fcvm.2026.1839483

**Published:** 2026-09-04

**Authors:** Satoshi Yasuda, Hayato Tada, Junya Ako

**Affiliations:** 1 Department of Cardiovascular Medicine, Tohoku University Graduate School of Medicine, Sendai, Miyagi, Japan; 2 Department of Cardiovascular Medicine, Kanazawa University Graduate School of Medical Sciences, Kanazawa, Ishikawa, Japan; 3 Department of Cardiovascular Medicine, Kitasato University, Sagamihara, Kanagawa, Japan

**Keywords:** atherosclerosis, atherosclerotic cardiovascular disease, biomarkers, inflammation, interleukin-6

## Abstract

Atherosclerotic cardiovascular disease (ASCVD) is a chronic inflammatory disease characterized by plaque formation. Inflammatory activity, assessed by high-sensitivity C-reactive protein (hsCRP), is linked to increased ASCVD risk. Recently, there has been much interest in the association between interleukin-6 (IL-6), a cytokine lying upstream of CRP along the inflammatory pathway, and ASCVD. Pre-clinical studies have shown that targeting IL-6 via its mediators may reduce pro-inflammatory effects. In observational studies, IL-6 has been identified as a source of residual risk for ASCVD even after controlling for factors such as male sex, old age and prior ASCVD events. Both genetic predisposition and acquired conditions, such as chronic systemic inflammation and comorbidities, may contribute to modest but persistent elevation in IL-6 levels. While routine IL-6 testing may not currently be feasible in practice, measurements may be informative in selected high-risk patients, as IL-6 may reflect inflammatory activation at an earlier stage than hsCRP. Detecting subtle yet clinically meaningful increases in inflammatory biomarkers in high-risk patients could facilitate earlier identification of ASCVD risk. Additionally, current evidence supports IL-6 as a potential therapeutic target for ASCVD. While trials investigating IL-6 inhibition specifically in ASCVD are limited, studies in patients with autoimmune conditions at increased cardiovascular risk have shown IL-6 inhibition to reduce inflammatory biomarkers without an excess of major adverse cardiovascular events. However, as IL-6 inhibition may raise triglyceride and low-density lipoprotein cholesterol levels, both of which are recognized ASCVD risk factors, further studies are needed to clearly assess the efficacy and safety of IL-6 inhibitors in ASCVD.

## Introduction

Atherosclerotic cardiovascular disease (ASCVD) is a chronic inflammatory disease encompassing coronary artery disease (CAD), peripheral artery disease, cerebrovascular disease and aortic atherosclerosis (1, 2). ASCVD is characterized by plaque formation within the arteries, which is driven by an increased concentration of blood cholesterol, particularly low-density lipoprotein cholesterol (LDL-C) (3). Accumulated LDL-C in the arteries may become oxidized, which in turn acts as a chronic stimulator of the innate and adaptive immune response (3). Stimulation of immune responses induces endothelial cells and smooth muscle cells to express adhesion molecules that lead to plaque progression and recruitment of macrophages as part of a pro-inflammatory response (3). Atherosclerotic plaques can obstruct coronary blood flow and cause symptoms such as stable angina, or may rupture or erode, resulting in complications such as thrombosis (3).

Given the wide-ranging clinical manifestations of atherosclerosis, ASCVD remains a significant global health challenge. In 2019, there was an estimated total of 197.22, 77.19 and 113.44 million cases of ischemic heart disease, ischemic stroke and peripheral artery disease, respectively (4). Earlier onset of ASCVD, demonstrated by an increasing prevalence of ASCVD among younger adults (20–54 years) by up to 20.55% from 1990 to 2019 (4), is particularly concerning as it highlights a potential increase in the accrued burden of ASCVD.

Older age, male sex, non-Hispanic Black ethnicity, smoking, alcohol consumption, obesity, diabetes, hypertension, elevated LDL-C or triglycerides and prior ASCVD events are well-recognized risk factors for ASCVD (1, 5). A combination of pharmacologic and non-pharmacologic interventions targeting or addressing these risk factors are hallmarks in the management of ASCVD (6–8). Typically, the first-line pharmacologic treatment option for the primary prevention of ASCVD is statins (7–9); statins reduce blood LDL-C levels, a key driver of plaque formation (10), and may also have pleiotropic effects (10, 11), such as reducing the levels of inflammatory biomarkers (12–14), due in part to their LDL-C lowering effect (12). In advanced cases, interventions such as percutaneous coronary intervention or coronary artery bypass grafting may be required depending on the patient's symptoms and imaging findings (9), even though these procedures may increase acute inflammation (15).

Recent advancements in ASCVD therapeutics suggest that inflammatory pathways involved in the pathogenesis of ASCVD may be viable targets in treatment (16). Mechanistically, inflammation plays a key role in the development and progression of atherosclerotic plaque and modulation of plaque formation, growth and stability (5, 17). The potential utility of monitoring levels of inflammation using biomarkers such as C-reactive protein (CRP), interleukin(IL)-6, IL-1β and IL-10 further suggests that these could be beneficial in the management and/or treatment of ASCVD (18).

To the authors' knowledge, there has been particular interest in IL-6, perhaps facilitated by existing access to and familiarity with drugs targeting IL-6 in clinical practice and evidenced by the abundance of literature published on IL-6. Discovered by a Japanese research group in 1986 (19), IL-6 is a pleiotropic cytokine secreted by macrophages, vascular smooth muscle and endothelial cells in the arterial wall (20, 21). IL-6 acts through three signaling modes: classic, trans- and cluster signaling (21, 22). In classic signaling, IL-6 binds to the membrane-bound IL-6 receptor (IL-6R) on hepatocytes and immune cells; this pathway is an important driver of acute-phase immune responses, and also potently induces production of CRP (21, 23). For cells not expressing membrane-bound IL-6R, IL-6 initiates trans-signaling by forming a complex with soluble IL-6R, and subsequently binds to glycoprotein 130 (19, 21–23). This leads to a pro-inflammatory effect, as IL-6 promotes cell adhesion and results in the development of atherosclerotic plaques (20). In cluster signaling, also known as trans-presentation, a cell-to-cell mechanism occurs where an IL-6-IL-6R complex engages glycoprotein 130 on T cells, promoting the differentiation and pro-inflammatory activity of pathogenic T helper 17 (T_H_17) cells (19, 22).

### Objectives

Inflammation plays a key role in the development and progression of atherosclerotic plaques and there is growing interest in IL-6 as an inflammatory biomarker in the management of ASCVD. Therefore, based on published literature and expert opinion, this review aims to provide a comprehensive summary and discussion of the role of IL-6 in ASCVD pathophysiology, highlighting any potential differences in IL-6 levels, such as ethnicity and lifestyle behaviors, as well as their implications on ASCVD management.

## Methods

Targeted searches based on a pre-specified protocol were conducted iteratively to identify pre-clinical, observational and clinical trial studies published after 2004, relevant to the discussion of IL-6 and ASCVD. Searches were done in PubMed and the first 50 results from each search strategy were evaluated against pre-defined eligibility criteria for relevance.

To capture the most recent advancements in research, supplementary searches of congress proceedings were conducted for literature published by the following congresses over the last two years (2023–2024): American College of Cardiology, American Heart Association and European Society of Cardiology (ESC).

All search strategies were recorded (Supplementary Material 1) and an inventory of the relevant literature identified was developed. Following peer review, the reference list was further supplemented with two additional relevant studies identified by a reviewer. Quality assessments were not conducted on studies included, as the objective of the targeted searches was to identify studies relevant to this narrative synthesis, rather than a comprehensive, formal analysis of the literature.

### Ethical approval

The targeted searches performed were based on published data or existing literature. As no patients or prospective patient-level data were involved in the searches, ethical approval was not required.

## IL-6 and ASCVD

**Figure 1:**
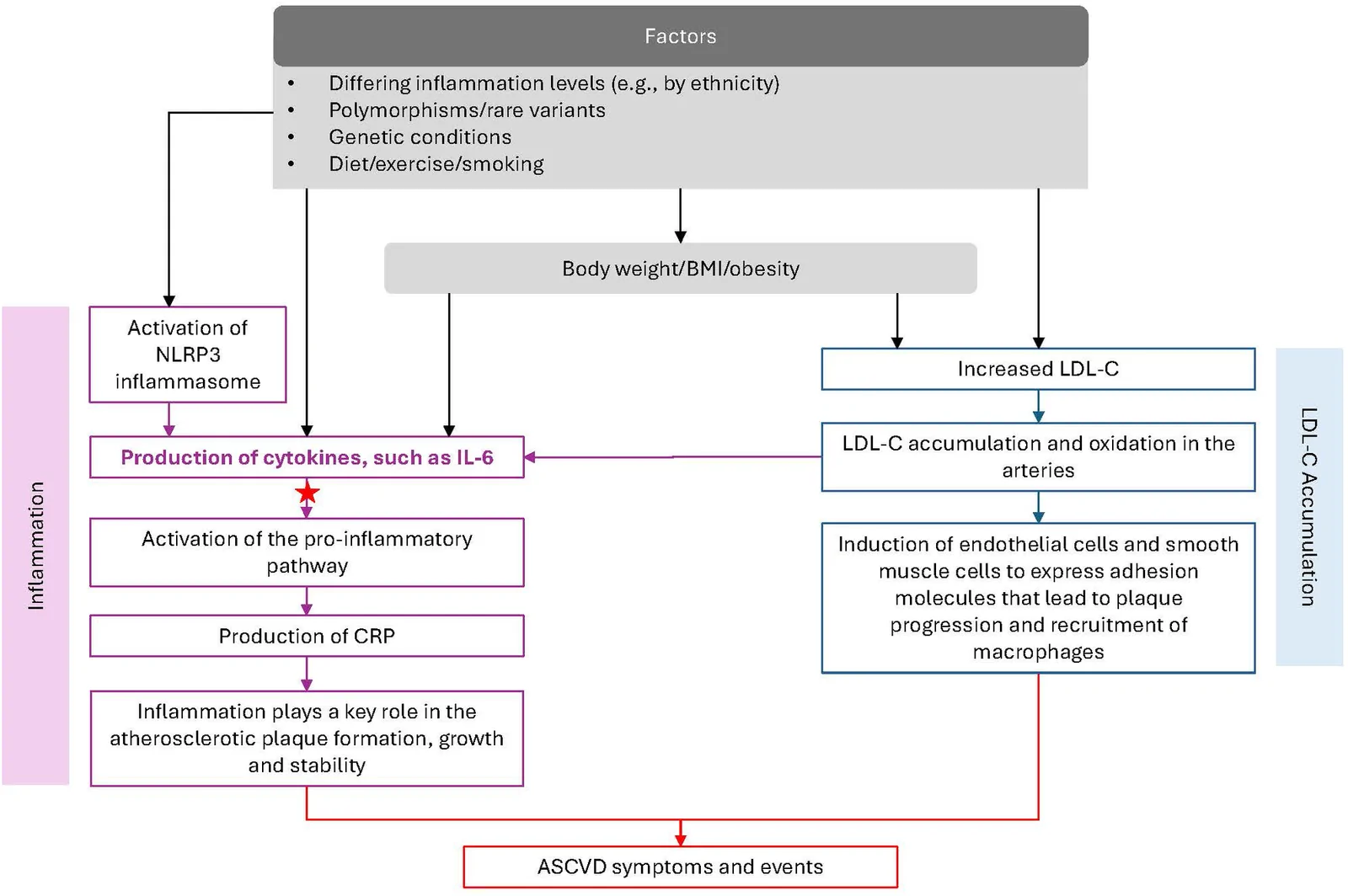
Relationship of factors affecting IL-6 and LDL-C in ASCVD and potential for targeting IL-6 in ASCVD management. Grey boxes depict potential factors impacting inflammation and LDL-C levels, pink boxes depict inflammation and blue boxes depict LDL-C accumulation. Arrows depict a flow or connection. The star represents IL-6 or IL-6R inhibition. ASCVD, atherosclerotic cardiovascular disease; BMI, body mass index; CRP, C-reactive protein; IL-6, interleukin-6; LDL-C, low-density lipoprotein cholesterol; NLRP3, NOD-like receptor family pyrin domain-containing 3.

A summary of findings linking IL-6 to ASCVD is presented in Figure 1; key factors or processes affecting levels of IL-6 are shown to demonstrate their interplay and potential culmination in ASCVD symptoms and/or events.

### Pre-clinical and mechanistic/genetic findings

While IL-6 itself exerts its effects on the inflammatory pathway by regulating the production of other inflammatory proteins, its expression may be modulated by other genes and products along this cascade (24, 25). Pre-clinical studies have shown that both upstream as well as downstream mediators are involved in modulating IL-6 expression (26–30), suggesting that these mediators may be potential molecular targets in reducing IL-6 to manage ASCVD risk.

Mendelian randomization studies provide a critical link between these mechanistic observations and clinical evidence by supporting a causal role for IL-6 in ASCVD. Mendelian randomization studies have demonstrated both that downregulating IL-6 production or inhibiting IL-6R may lower the risk of ASCVD on top of lipid-lowering effects (24, 25). One such study showed that up to 70 circulating proteins involved in cytokine production and regulation or immune cell recruitment and differentiation were significantly affected by IL-6 production (25). These circulating proteins were associated with increased risk of CAD, such as the C-X-C motif chemokine ligand 10 (25). Therefore, similar to how Mendelian randomization studies in dyslipidemia eventually shifted clinical practice regarding LDL-C management by showing that LDL-C is a causal factor for ASCVD while HDL-C is not (31), these Mendelian randomization studies provide a strong foundation for informing changes in clinical practice regarding IL-6 in ASCVD. As targeting the IL-6 signaling pathway through IL-6 or IL-6R genetic instruments was also associated with a similar risk reduction for multiple cardiometabolic diseases, this further supports that IL-6 may be a potential therapeutic target for cardiovascular diseases, beyond its role as a biomarker (16).

Additionally, upstream mediators of IL-6 along the inflammatory pathway have also been investigated (26–28), as they could present as potential therapeutic targets. Studies in mouse models identified genetic drivers of mediators such as adenosine deaminases acting on RNA-1/-2, which decreased IL-6 levels and reduced both atherosclerotic plaque burden and leukocyte infiltration in atherosclerotic plaques (28). Another gene, Mettl14, was found to upregulate IL-6 expression, thereby modulating the inflammatory state of macrophages in atherosclerosis, upregulating the pro-inflammatory response and finally, increasing the formation of atherosclerotic plaque (27). Data presented at the American Heart Association 2024 congress showed that knocking out the Dnmt3a gene in mouse models blunted atherogenesis by stimulating the IL-6 classical signaling pathway and led to an anti-inflammatory effect (30). Other than genetic drivers of IL-6, circulating micro ribonucleic acid (miRNA) has also been associated with upregulation or downregulation of IL-6 production; non-wild-type macrophages modified to knock out miRNA-223 significantly increased IL-6 levels compared to wild-type macrophages, indicating that miRNA-223 suppressed pro-inflammatory activation by macrophage cells (26). Alternatively, the effect of IL-6 on ASCVD may also be regulated via downstream mediators. A study presented at the 2024 ESC congress found that treating mice with anti-IL-6 antibodies reduced platelet reactivity to collagen, which decreased arterial thrombosis upon endothelial damage (29).

These pre-clinical studies suggest that a pro-inflammatory state may be minimized by targeting IL-6 or its pathways. Taken together with causal evidence from Mendelian randomization studies, the authors posit that these mechanistic findings have strong translational relevance to clinical application. The clinical relevance and implications of these mechanisms may be further elucidated from observational studies exploring the relation between IL-6 and ASCVD in patients in clinical practice.

### Observational findings

Complementing findings from pre-clinical studies, several observational studies have further investigated the impact of IL-6 for both primary or secondary prevention of ASCVD in clinical practice (2, 32–38). These have identified inflammation as a source of residual risk for ASCVD after controlling for other risk factors such as body mass index (BMI), prior MI, prior stroke and ethnicity (2, 34, 39, 40). The findings corroborate that the association between IL-6 and ASCVD at a molecular level were similarly observed at a clinical level, while elucidating a range of potential genetic and acquired factors affecting IL-6 levels (28, 40–49).

A number of recent studies have reported significant associations between IL-6 and ASCVD. In a single-site biomarker study of 478 patients from the United States (US), those who experienced ASCVD events such as acute coronary syndrome (ACS) had IL-6 levels almost twice as high as those who did not (32). Furthermore, separate studies have found significant associations between higher IL-6 concentrations and overall cardiovascular disease risk (including ASCVD), all-cause mortality and cardiovascular mortality (including those caused by ASCVD), even after adjusting for demographic characteristics (2, 33, 34). One multicenter cohort study conducted in Switzerland showed that in patients with acute myocardial infarction (MI), those with higher IL-6 levels demonstrated significantly higher rates of death or recurrent acute MI within 1 year, with early divergence of the Kaplan–Meier curves suggesting that IL-6 may be a valuable target for early anti-inflammatory interventions (35). Multiple cohort studies have also found both high-sensitivity CRP (hsCRP) and IL-6 to be significantly associated with cardiovascular risk (2, 50); however, while the association between hsCRP and cardiovascular risk was no longer significant after adjusting for IL-6, IL-6 maintained a significant association with cardiovascular risk even after adjusting for hsCRP (2, 38, 50). Thus, in clinical practice, IL-6 may have a greater prognostic value than hsCRP to detect primary cardiovascular events in adults.

Specifically related to laboratory or imaging measures of ASCVD, studies have demonstrated that higher IL-6 levels were significantly associated with carotid plaque number, fibrinogen level, aortic valve calcifications, higher pulmonary artery pressure, left atrial diameter and ST-elevated MI (36, 37). These conditions and events can promote atherosclerosis by increasing the number of calcifications and plaques, leading to disease progression (36).

An international online survey presented at the 2024 ESC congress showed that 58% of surveyed cardiologists indicated that findings on systemic inflammation in patients with ASCVD would not change their treatment plan, although 64% agreed that systemic inflammation is one of the key drivers for cardiovascular events in patients with ASCVD and chronic kidney disease (CKD), both of which are chronic inflammatory conditions (51). Nevertheless, 62% of the surveyed cardiologists would like to better understand the role of systemic inflammation in ASCVD (51), which highlights a potential key unmet need relevant to the understanding of ASCVD and its management. Therefore, systemic inflammation may be a valuable consideration in ASCVD management, particularly due to the differing patient profiles and presentations of ASCVD, which may be driven by both genetic and acquired factors.

### Observational and clinical trial findings on ethnicity, geography and acquired factors impacting IL-6

IL-6 levels have been found to vary across geography and ethnicity (2, 34, 52). For example, levels of IL-6 were reportedly significantly lower in Japanese [mean ± standardized difference (SD): 1.70 ± 1.99 pg/mL, *p* < 0.01] than in Caucasians and African-Americans (mean ± SD: 2.79 ± 2.30 pg/mL and 4.16 ± 3.72 pg/mL, respectively) from a US population-based study (52). Separately, in an international randomised controlled trial among patients with coronary heart disease, IL-6 levels varied significantly at baseline (i.e., before being treated with the intervention they were randomised to: darapladib or placebo) by geographic region and race (42). The proportion of patients with high baseline IL-6 (≥3.2 ng/L) was highest in South America (35.9%) and lowest in Asia-Pacific (20.8%) (42). When stratified by race, East Asians had the lowest proportion of patients with high baseline IL-6 (15.6%) and Black patients had the highest proportion (35.8%) (42). Although these findings should be interpreted with caution due to the lack of standardized assays and potential differences in sample collection and analytical methods, which limits the direct comparison of IL-6 concentrations across studies, ethnic variations may nevertheless have arisen due to genetic differences; for instance, IL-6 polymorphisms such as rs1800795 have been associated with greater susceptibility to CAD in Asians (41).

Acquired factors, such as lifestyle behaviors (e.g., smoking, diet and exercise) and presence of specific comorbidities, may also increase circulating IL-6 (43, 48, 49, 52). By elevating IL-6 levels, these acquired factors can upregulate the pro-inflammatory pathway, resulting in increased hsCRP levels and a subsequent significantly increased risk of ASCVD events such as MI and hospitalization for heart failure (42). Smoking, for example, is a known contributor to inflammation and significantly increased levels of IL-6 have been found in heavy smokers, compared with healthy volunteers, causing a pro-inflammatory effect (43). Diet may also influence IL-6 levels, as shown by decreased levels of circulating IL-6 associated with the adoption of reduced calorie diets (e.g., nut-enriched low-calorie diet, intermittent fasting) (45, 47). Furthermore, introducing and maintaining exercise routines, such as high-interval intensity training, may reduce levels of IL-6 and potentially reduce chronic inflammation (48, 49). Finally, comorbidities such as type 2 diabetes mellitus (T2DM) may also contribute to chronic inflammation and elevate IL-6 levels (49). Lifestyles and behaviors, which in turn can contribute to the prevalence of comorbidities such as T2DM or obesity, are influenced by various environmental, psychosocial and cultural factors (52). For example, a typical Japanese diet tends to differ substantially from western-style diets, which can impact body weight and thus underlie the association between ethnicity and IL-6 levels (52). Indeed, studies have suggested that ethnic differences in IL-6 may be partially attributed to BMI, which is itself independently linked to IL-6 (46), and can account for over 7% of variance in circulating IL-6 levels (44).

Despite variations in IL-6 levels across population subgroups as outlined above, significant associations between IL-6 and ASCVD have been repeatedly observed for most cardiovascular outcomes across ethnicities, including cardiovascular mortality due to ASCVD (2, 34). Given the association between IL-6 and ASCVD risk, a more complete understanding of the differences in IL-6 by ethnicity would thus be important to inform clinical decision-making for ASCVD management.

### Clinical trial findings on potentially targeting IL-6 for ASCVD management

Drugs that inhibit IL-6 and IL-6R are available for the management of chronic conditions such as rheumatoid arthritis (RA), but are not currently indicated for ASCVD. While there have been clinical trials assessing the efficacy and cardiovascular safety of IL-6 inhibitors and IL-6R inhibitors in specific chronic conditions, trials specifically assessing these aspects in patients with ASCVD are limited. Table 1 summarizes trial findings of IL-6 inhibitors or IL-6R inhibitors that report measures or outcomes related to ASCVD.

**Table 1:** List of trials on IL-6 or IL-6R inhibitors reporting outcomes related to ASCVD.

Trial name	Study design	Status	Patient population	Treatment arms	Cardiovascular-related clinical outcomes reported	Inflammatory biomarkers reported	Lipid levels reported
Kleveland 2016 (NCT01491074) (53)	Double-blind, randomized placebo-controlled phase 2 trial	Completed (2011–2014)	Adults with NSTEMI	Tocilizumab 280 mg Placebo	Not reported	Median AUC for hsCRP during 3-day hospitalization was 2.1 times higher in the placebo than in the tocilizumab group (4.2 vs. 2.0 mg/L/h, *p* < 0.001) In the placebo group, there was a modest but significant increase in IL-6 during 3-day hospitalization; in the tocilizumab group, the increase in IL-6 was pronounced and persisted during hospitalization. Between-group differences in changes from baseline were significant throughout 3-day hospitalization IL-6R did not change in the placebo group, whereas a substantial, gradual increase in IL-6R was observed during 3-day hospitalization in patients allocated to tocilizumab No between-group differences in IL-6 parameters at 3 and 6 months	Not reported
ENTRANCE (NCT01331837) (54)	Open-label, randomized, phase 4 trial	Completed (2011–2016)	Adults with moderate-to-severe RA who are inadequate responders to ≥1 non-biologic DMARD and ≥1 traditional CVD risk factor, extraarticular RA manifestations, or history of a CVD event	Tocilizumab 8 mg/kg Etanercept 50 mg	83 MACEs in tocilizumab arm Tociluzumab had a higher MACE HR than etanercept, but this was not statistically significant	Not reported	Tocilizumab had significantly greater increases in LDL-C (median 11.1% greater increase), HDL-C (median 5.4% greater increase) and triglyceride levels (median 13.6% greater increase) compared to etanercept
STAT-MI (NCT02419937) (55)	Double-blind, randomized, placebo-controlled trial	Completed (2015–2017)	Adults with evidence of an acute MI (including NSTEMI and STEMI)	Tocilizumab 162 mg Placebo	9 MACEs in the tocilizumab arm, but this was not significantly more than the placebo arm (3 MACE)	Non-statistically significant increase in CRP in tocilizumab arm after receiving medication, compared with placebo	Not reported
ASSAIL-MI (NCT03004703) (56–59)	Double-blind, randomized placebo-controlled phase 2 trial	Completed (2017–2021)	Adults admitted to hospital due to acute STEMI	Tocilizumab 280 mg Placebo	Adjusted myocardial salvage index (MSI) was higher in the tocilizumab arm than in the placebo arm	AUC of CRP during hospitalization was substantially lower in the tocilizumab group than in the placebo group (*p* < 0.001) IL-6 signalling pathway was downregulated in the tocilizumab arm, compared to patients allocated to placebo, after 3–7 days IL-6R expression at hospitalization was inversely correlated with MSI	Low-density lipoprotein and triglycerides increased in the tocilizumab arm compared with the placebo arm (significant between-group difference at 24 and/or 168 h) No between-group differences at 3 and 6 months
EXTEND (NCT01146652) (60)	Open-label, non-randomized, phase 3 trial	Completed (2010–2020)	Adults with RA who were inadequate responders, intolerant or failed treatments with MTX, anti-TNF agents or non-biologic DMARDs	Sarilumab 200 mg	Exposure-adjusted incidence rates of MACE ranged from 0.2 to 0.6 per 100 patient years, depending on the background treatment received in the parent trials	Not reported	Not reported
SIRROUND LTE (NCT01856309) (61)	Open-label LTE study of RCTs	Completed (2013–2018)	Adults with moderate-to-severe RA who were refractory to DMARDs and anti-TNF agents	Sirukumab 50 mg Sirukumab 100 mg	29 MACEs in the sirukumab arms	Not reported	Sirukumab was associated with numerical changes in total cholesterol, LDL-C (up to 69.9% increased to >130 mg/dL), HDL-C (up to 16.1% decreased to <40 mg/dL) and fasting triglycerides (up to 34.9% increased to >250 mg/dL) at Week 52 among patients with normal levels at baseline
MONARCH OLE (NCT02332590) (60)	Open-label, randomized, phase 3 trial	Completed (2016–2020)	Adults with RA deemed inappropriate candidates, intolerant or inadequate responders to MTX	Sarilumab 200 mg	Exposure-adjusted incidence rates of MACE ranged from 0.2 to 0.6 per 100 patient years, depending on the background treatment received in the parent trials	Not reported	Not reported
CREDO4 (NCT03120949) (62)	Open-label, randomized, phase 3 RCT	Completed (2017–2021)	Adults with active RA not responding adequately to MTX or a TNF inhibitor who completed the CREDO parent trials	Olokizumab 64 mg (every 2 weeks) Olokizumab 64 mg (every 4 weeks)	8 cardiovascular deaths across both olokizumab arms 5 non-fatal MI across both olokizumab arms 12 non-fatal stroke/TIA across both olokizumab arms 3 non-fatal coronary revascularization procedures across both olokizumab arms	Olokizumab was associated with decreased lipoprotein(a) (up to mean −23.63 nmol/L at Week 24)	Olokizumab was associated with increases in LDL-C (up to 9.1% increase to ≥160 mg/dL) and HDL-C (up to 29.5% increased to ≥60 mg/dL) and triglycerides (up to 28.6% increased to ≥200 mg/dL) among patients with low levels at baseline Elevation of blood lipids was reported as an AE in a dose-dependent rate of 14.94/100 patient years for olokizumab every 2 weeks and 11.94/100 patient years for olokizumab every 4 weeks
RESCUE (NCT03926117) (63)	Double-blind, randomized, placebo-controlled phase 2 RCT	Completed (2019–2020)	Adults with stage 3–5 CKD with residual inflammatory risk (hsCRP ≥2 mg/dL)	Ziltivekimab 7.5 mg Ziltivekimab 15 mg Ziltivekimab 30 mg Placebo	1 cardiovascular death in placebo arm 1 non-fatal MI in ziltivekimab 7.5 mg arm 1 non-fatal MI in ziltivekimab 15 mg arm	Ziltivekimab was associated with significant reductions in hsCRP (up to 92%) compared to placebo Ziltivekimab was associated with significant dose-dependent reductions for fibrinogen (up to 37%) and SAA (up to 50%) compared to placebo Ziltivekimab significantly reduced levels of lipoprotein(a) (up to 25.0%) compared to placebo	Ziltivekimab led to significant increases in triglycerides (up to 8%) compared to placebo Ziltivekimab led to numerical increases in LDL-C (up to 5%) compared to placebo Ziltivekimab led to significant increases in HDL-C (up to 14%) but there was no substantive shift in total cholesterol to HDL-C ratio compared to placebo
RESCUE-2 (NCT04626505) (64)	Double-blind, randomized, placebo-controlled phase 2 RCT	Completed (2020–2021)	Japanese adults with stage 3–5 CKD with residual inflammatory risk (hsCRP ≥2 mg/dL)	Ziltivekimab 15 mg Ziltivekimab 30 mg Placebo	Not reported	Ziltivekimab was associated with significant reductions in hsCRP (up to 96.2%) compared to placebo Ziltivekimab was associated with significant reductions for fibrinogen (up to 38.0%) and SAA (up to 71.4%) compared to placebo	Ziltivekimab was associated with numerically small increases from baseline to study end in LDL-C (up to 17.9%) Ziltivekimab was associated with numerically small increases from baseline to study end in HDL-C (up to 7.9%) but there was no significant change in total cholesterol to HDL-C ratio Ziltivekimab was associated with significant increases in triglyceride levels compared to placebo (up to 36.3% increase)
ZEUS (NCT05021835) (65)	Double-blind, randomized, placebo-controlled phase 3 RCT	Active, not recruiting (2021)	Adults with CKD and evidence of ASCVD	Ziltivekimab 15 mg Placebo	No results available	No results available	No results available
POSIBIL6ESKD (NCT05485961) (66)	Double-blind, randomized, placebo-controlled phase 2b/3 RCT	Recruiting (2022)	Adults with ESKD undergoing maintenance dialysis for at least 12 weeks and diagnosed diabetes or ASCVD	Clazakizumab 2.5 mg Clazakizumab 5 mg Clazakizumab 10 mg Placebo	Not reported	Clazakizumab was associated with significant reductions in hsCRP (up to 92%) compared to placebo Clazakizumab was associated with significant reductions for fibrinogen (up to 43%), SAA (up to 66%) and lipoprotein(a) (up to 52%) compared to placebo	Clazakizumab was associated with numerically small increases in total cholesterol (up to median 16.4 mg/dL), HDL (up to 4.8 mg/dL), LDL-C (up to 13.1 mg/dL) and triglycerides (up to 27.5 mg/dL) compared to placebo

AE, adverse event; ASCVD, atherosclerotic cardiovascular disease; AUC, area under the curve; CKD, chronic kidney disease; DMARD, disease-modifying anti-rheumatic drug; ESKD, end-stage kidney disease; HDL-C, high-density lipoprotein cholesterol; hsCRP, high-sensitivity C-reactive protein; IL-6, interleukin-6; LDL-C, low-density lipoprotein cholesterol; MACE, major adverse cardiovascular event; MI, myocardial infarction; MSI, myocardial salvage index; MTX, methotrexate; NSTEMI, non-ST-elevation myocardial infarction; RA, rheumatoid arthritis; RCT, randomized controlled trial; SAA, serum amyloid A; STEMI, ST-elevation myocardial infarction; TIA, transient ischemic attack; TNF, tumour necrosis factor.

Clinical trials of IL-6 inhibitors and IL-6R inhibitors in RA suggest that these drugs are generally well-tolerated in patients at risk of cardiovascular disease, in both primary and secondary prevention settings (54, 60, 61, 66). For example, the IL-6/IL-6R inhibitors sarilumab and sirukumab were associated with minimal major adverse cardiovascular events (MACEs) in clinical trials for RA (60, 61). Additionally, among patients receiving dialysis who had cardiovascular disease or diabetes, clazakizumab significantly decreased levels of hsCRP after 12 weeks of treatment, compared to placebo (66). Overall, the findings indicate that inhibition of IL-6 or IL-6R did not appear to negatively affect cardiovascular safety in patients with systemic inflammation who are at risk of primary or secondary cardiovascular disease.

Therefore, since IL-6 is a known biomarker of cardiovascular risk, predicting outcomes such as MI, heart failure and all-cause mortality (37, 67), it may be a potential therapeutic target for management of ASCVD. Phase 2 clinical trials in patients at high risk of primary or secondary atherosclerotic events (RESCUE; RESCUE-2) have shown that the IL-6 inhibitor ziltivekimab was well-tolerated and led to significant reductions in hsCRP compared to placebo (63, 64). Additionally, there were significant reductions in atherosclerotic biomarkers, such as fibrinogen, among patients who received ziltivekimab compared to placebo in US populations (RESCUE) (63). Similar significant reductions in hsCRP and fibrinogen were also observed among Japanese subgroups who had lower baseline inflammation levels than the American population in RESCUE (64). For, tocilizumab, an IL-6R antagonist, the STAT-MI trial reported no significant differences in MACEs with tocilizumab compared to placebo among patients with acute MI (55). Two phase 2 trials have been conducted in Norway investigating the use of tocilizumab in ST-elevation myocardial infarction (STEMI) and non-STEMI patients (53, 56–58). Kleveland et al., found significantly lower hsCRP but increased IL-6 and IL-6R levels in the tocilizumab group compared with placebo during 3-day hospitalization with NSTEMI (53). The authors posit that this was due to decreased IL-6R-mediated clearance of IL-6 caused by tocilizumab-mediated IL-6R blockade, given that the increased free IL-6 was accompanied by decreased CRP levels, indicating decreased IL-6 bioactivity (53). ASSAIL-MI similarly found significantly lower CRP during hospitalization in the tocilizumab group compared to placebo, with sub-studies investigating immune cell and monocyte profiles and noting that the IL-6 signaling pathway was downregulated in the tocilizumab arm vs. placebo (56), and that IL-6R expression was inversely associated with improvements in myocardial salvage index (57). Overall, the robust reduction in CRP demonstrated in the tocilizumab arm in ASSAIL-MI suggested powerful inhibition of the IL-6 pathway by tocilizumab (58).

These trials of sarilumab, sirukumab, clazakizumab, ziltivekimab and tocilizumab collectively suggest that inhibiting IL-6 or IL-6R may be beneficial in patients at risk of primary or secondary atherosclerotic events, given their safety and effect on the reduction of inflammatory and atherosclerotic biomarkers.

## Discussion/expert opinion

### Comparing biomarkers of inflammation in ASCVD

Current risk stratification models for cardiovascular disease, such as PREVENT or QRISK3 do not incorporate IL-6 or CRP levels; therefore, the ability of these models to discriminate risk remains incomplete (68, 69). Rather than viewing CRP and IL-6 as mutually exclusive biomarkers, the authors note that the two inflammatory biomarkers may provide additional value for both primary and secondary ASCVD risk stratification. This is especially as these biomarkers have been shown to represent components of residual cardiovascular risk even in patients receiving optimal medical therapy, such as statins (70). Therefore, on top of conventional risk stratification models, physicians may want to consider CRP and IL-6 monitoring during the diagnostic management of patients.

In its 2024 guidelines for chronic coronary syndrome, the ESC currently recommends clinicians to consider hsCRP in the initial diagnostic management of patients suspected of chronic coronary syndrome, since it is a recognized predictor for future cardiovascular events (9). At the same time, the 2025 update on dyslipidemia management in cardiovascular risk reduction highlights persistently elevated hsCRP (>2 mg/L) as a risk modifier of cardiovascular disease (without providing a specific definition for “persistent”) and suggests that clinicians consider hsCRP levels in determining a patient's risk category (71). Nevertheless, despite hsCRP being a more commonly used inflammatory biomarker at present, recent studies suggest that IL-6 may be a valuable alternative inflammatory biomarker for ASCVD management (42, 72–77).

IL-6 could be a more sensitive predictor of cardiovascular events than hsCRP (72, 73). As IL-6 acts upstream of the inflammatory cascade, it may therefore reflect vascular inflammation and injury at an earlier stage, potentially before hsCRP levels increase (72). A study comparing predictive abilities for ischemic heart disease found that hsCRP and IL-6 were two inflammatory markers with the highest predictive abilities (76). Similarly, other observational studies generally reported IL-6 to be similar or significantly better predictors for cardiovascular outcomes (mortality, stroke severity, coronary artery calcium, atherosclerosis) than hsCRP or CRP (42, 73–75, 77). In some studies, hsCRP was no longer a significant predictor of cardiovascular events after adjusting for demographic characteristics and other known risk factors of ASCVD, while IL-6 remained a significant predictor (42, 74). Furthermore, although hsCRP was a more sensitive biomarker of long-term cardiovascular mortality compared to IL-6 (100% vs. 94%, respectively) in patients with ACS, hsCRP was much less specific than IL-6 (19% vs. 48%, respectively), suggesting that IL-6 could be a more valuable predictor to reduce false positives (72). Other inflammatory biomarkers from the same inflammatory cascade include the nucleotide-binding domain, leucine-rich-containing family, and pyrin domain-containing-3 (NLRP3) inflammasome. While NLRP3 has been abundantly researched for its mechanism and role in inflammation (78), it currently lacks therapeutic options compared to the availability of IL-6-targeting drugs. As such, IL-6 may be a more reliable biomarker and feasible therapeutic target to directly reduce inflammation and manage ASCVD risk.

### Clinical implementation of IL-6 as a biomarker

Given the associations between IL-6 and cardiovascular events, emerging data present IL-6 as not only valuable as a biomarker for assessing acute and chronic inflammation, but as an additional consideration to other recognized risk factors for ASCVD (e.g., increased weight, male sex, smoking, hypertension, non-Hispanic Black ethnicity) (1). A US observational study showed that while White patients demonstrated generally lower inflammation levels than Black patients, IL-6 mediated 20% of the ethnic disparity in stroke risk at age 55 between Black and White participants (79). Though further studies are required to confirm the role of IL-6 in mediating racial disparities in ASCVD risk, particularly among Asian patients, the US study highlights the importance of IL-6 as a biomarker for ASCVD.

Systemic inflammation, as a consequence of chronic autoimmune conditions, is a recognized risk factor for ASCVD (80). Among patients with RA, there was a significant association between IL-6 and cardiovascular events (coronary heart disease, stroke and heart failure) (33). Elevated IL-6 was also more common in patients with ulcerative colitis who also had ASCVD, compared to those with ulcerative colitis without ASCVD (81). CKD has similarly been associated with increased IL-6 as well as atherosclerotic markers (82). While CKD itself may elevate IL-6 levels, management options for CKD such as dialysis can also increase IL-6 levels (83). In light of the association between elevated IL-6 due to systemic inflammation and ASCVD, a phase 3 clinical trial (ZEUS) is currently ongoing to evaluate the efficacy and safety of the IL-6 inhibitor ziltivekimab on MACE among patients with CKD, established ASCVD and systemic inflammation (defined as hsCRP ≥2 mg/L) (65). Other than chronic autoimmune conditions, chronic inflammation can also be caused by metabolic conditions such as T2DM; accordingly, T2DM has also been linked to elevated IL-6 and ASCVD risk (84). Among diabetic patients in a Danish population-based study, elevated remnant cholesterol (a known risk factor of ASCVD) and low-grade inflammation (measured as elevated hsCRP), explained up to 34% of cardiovascular risk (84).

Therefore, particularly in patients with conditions where inflammatory markers may be measured to assess disease, clinicians may consider monitoring inflammation levels as part of the clinical management of primary or secondary ASCVD (85). Detecting small but clinically meaningful increases in sensitive inflammatory biomarkers such as IL-6 may be particularly valuable in patient populations with lower baseline inflammation levels (e.g., by region or ethnicity), for earlier identification of increased ASCVD risk.

An international online survey presented at the 2024 ESC congress showed that this understanding of elevated IL-6 as an important risk factor for ASCVD and recurrent ASCVD events is not currently reflected in clinical practice, as only 64% of cardiologists in the survey tested for systemic inflammation (51). The authors note some barriers that limit the implementation of inflammatory biomarker testing, particularly IL-6, in laboratory medicines, such as the limited availability of biomarker testing, as well as the lack of standardized assays and validated clinical cut-offs. Although evidence has demonstrated the good analytical performance and accuracy of currently available assays (86), guidelines on the standardization of assays and validated clinical cut-off values could encourage the clinical implementation of IL-6 monitoring.

### Targeting IL-6 for ASCVD

Beyond their main mechanisms of action, some existing therapies for ASCVD have reported pleiotropic effects on reducing inflammation. For example, while statins may reduce IL-6-mediated inflammation (14, 87), this may be attributed to the reduced hepatic production of pro-inflammatory LDL-C, rather than direct effects on the inflammatory pathway (12, 88). Non-statin therapies, such as anti-thrombotics (e.g., aspirin, rivaroxaban), have also demonstrated some reduction in IL-6 levels among patients with ASCVD (e.g., previous MI or CAD) (89). More specifically in patients with T2DM and ASCVD, glucagon-like peptide-1 receptor agonists are a recommended treatment option (90), with evidence supporting its anti-inflammatory effects (e.g., decreased IL-6) (91) and reduction in ASCVD events (92). However, although such reductions in inflammation represent beneficial pleiotropic effects, these treatments do not directly target specific underlying drivers of inflammation (88, 89). This was apparent from findings of a systematic review where statins decreased hsCRP levels without producing significant changes in IL-6 concentrations (70).

Given the limitations of existing therapies in targeting inflammatory biomarkers such as IL-6, patients may remain at risk for ASCVD events despite active ASCVD treatment (70), which highlights a key area of unmet need. An international survey of cardiologists showed similar sentiments, where 57% identified the lack of treatment options as the greatest unmet need in patients with ASCVD and systemic inflammatory conditions such as chronic kidney disease (51). From this same survey, 49% of cardiologists believed that no existing medications were available to treat systemic inflammation specifically (51), despite evidence suggesting that existing ASCVD treatments can reduce inflammation to some extent. Access to suitable treatment options targeting underlying drivers of inflammation in ASCVD would therefore be a key advancement in this field.

To that end, new therapies that directly target systemic inflammation and are safe to use among patients at risk of ASCVD would need to be identified to address this unmet need. Such alternatives may be particularly valuable in patients who may not tolerate existing therapies, such as statins, which target LDL-C and are generally the first-line therapy recommended for reducing ASCVD risk (8). Similar to LDL-C, inflammatory biomarkers may be predictive of future ASCVD events such as MI, stroke, coronary revascularization and cardiovascular mortality (93). Hence, IL-6 presents as a promising efficacious target for ASCVD prevention and management; its potentially greater sensitivity and specificity as an inflammatory biomarker compared with hsCRP further supports this proposition (72). For example, evidence from the phase 2 RESCUE and RESCUE-2 trials of the IL-6 inhibitor ziltivekimab showing good tolerability and significant reductions in IL-6 in patients at risk of ASCVD are promising (63, 64). Phase 2 trials in Norway investigating the IL-6R antagonist tocilizumab have also demonstrated that it significantly attenuates inflammatory responses in STEMI and NSTEMI patients, based on significantly decreased CRP levels (53, 56–59). Reducing inflammation by direct inhibition of IL-6 could thus be an alternative avenue to improve the prognosis of patients at high risk of primary or recurrent ASCVD events.

However, although IL-6 inhibitors may be generally well-tolerated among patients at high risk of ASCVD, increased lipid levels have been reported in clinical trials. Specifically, in patients at increased risk of cardiovascular or ASCVD events, certain IL-6 inhibitors (sirukumab, clazakizumab and ziltivekimab) have shown small numerical increases in total cholesterol and LDL-C and significant increases in triglycerides (61, 63, 64, 66). As elevated cholesterol levels can contribute to atherosclerotic plaque formation, the risk-benefit profile of IL-6 inhibitors in ASCVD management should be carefully assessed in future research in order to ensure that IL-6 inhibition can be a suitable approach to safely manage ASCVD risk. Currently, no inhibitors of IL-6 or IL-6R are approved specifically for reducing cardiovascular risk in ASCVD. However, growing evidence and interest in the role of IL-6 in ASCVD suggests that there are benefits to exploring IL-6 inhibitors or IL-6R inhibitors in future clinical trials as potential therapeutic options for the chronic management of ASCVD (9).

## Conclusion

Published evidence shows IL-6 as a source of residual risk and its potential role as a prognostic factor for ASCVD. While IL-6 levels may vary across populations, the association between IL-6 and ASCVD remains significant across ethnicities. As IL-6 may be a more sensitive and upstream biomarker of systemic inflammation compared to hsCRP, IL-6 levels may be valuable to assess patients' ASCVD risk and management plan. Especially in patient populations with lower baseline inflammation levels (e.g., by region or ethnicity) or at high risk of ASCVD, small but clinically meaningful increases in IL-6 detectable through IL-6 testing may be valuable for earlier ASCVD risk identification. The potential of IL-6 inhibitors or IL-6R inhibitors to reduce systemic inflammation among patients with ASCVD suggests that they may be considered alongside existing ASCVD treatments. However, further research is needed to clarify the overall impact of IL-6 inhibition on lipid metabolism and to fully evaluate the efficacy and safety of such therapies in ASCVD.
